# Duration of Invasive Mechanical Ventilation before Veno-Venous ExtraCorporeal Membrane Oxygenation for Covid-19 related Acute Respiratory Distress Syndrome: The experience of a tertiary care center

**DOI:** 10.1016/j.heliyon.2024.e31811

**Published:** 2024-05-29

**Authors:** Charles Vidal, Mathilde Nativel, Bérénice Puech, Florent Poirson, Radj Cally, Laurence Dangers, Eric Braunberger, Julien Jabot, Nicolas Allou, JérômeM Allyn

**Affiliations:** a Service de Réanimation polyvalente, Centre Hospitalier Universitaire Felix Guyon Allée des Topazes, 97405, Saint Denis, France; b Service de chirurgie cardio-thoracique et vasculaire, Center Hospitalier Universitaire Felix Guyon Allée des Topazes, 97405, Saint Denis, France

**Keywords:** VV-ECMO, Covid-19, ARDS, Duration of invasive mechanical ventilation

## Abstract

**Background:**

Veno-Venous Extracorporeal Membrane Oxygenation (VV-ECMO) is an efficient ventilatory support in patients with refractory Covid-19-related Acute Respiratory Distress Syndrome (ARDS), however the duration of invasive mechanical ventilation (IMV) before ECMO initiation as a contraindication is still controversial. The aim of this study was to investigate the impact of prolonged IMV prior to VV-ECMO in patients suffering from refractory Covid-19-related ARDS.

**Methods:**

This single-center retrospective study included all patients treated with VV-ECMO for refractory Covid-19-related ARDS between January 1, 2020 and May 31, 2022. The impact of IMV duration was investigated by comparing patients on VV-ECMO during the 7 days (and 10 days) following IMV with those assisted after 7 days (and 10 days). The primary endpoint was in-hospital mortality.

**Results:**

Sixty-four patients were hospitalized in the ICU for Covid-19-related refractory ARDS requiring VV-ECMO. Global in-hospital mortality was 55 %. Median duration of IMV was 4 [2; 8] days before VV-ECMO initiation. There was no significant difference in in-hospital mortality between patients assisted with IMV pre-VV-ECMO for a duration of ≤7 days (≤10 days) and those assisted after 7 days (and 10 days) ((*p* = 0.59 and *p* = 0.45).

**Conclusion:**

This study suggests that patients assisted with VV-ECMO after prolonged IMV had the same prognosis than those assisted earlier in refractory Covid-19-related ARDS. Therefore, prolonged mechanical ventilation of more than 7–10 days should not contraindicate VV-ECMO support. An individual approach is necessary to balance the risks and benefits of ECMO in this population.

## Highlights

• VV-ECMO is an efficient ventilatory support in patient with refractory ARDS.
• Duration of invasive mechanical ventilation (IMV) before ECMO as contraindication is controversial in Covid-19-related ARDS.
• Patients assisted after 7 or 10 days of (IMV) had the same prognosis as those assisted earlier.

## Abbreviations

ARDS: Acute Respiratory Distress Syndrome
BMI: Body Mass Index
CI: Confidence Interval
Covid-19: Coronavirus disease 2019
HFNO: High Flow Nasal Oxygenation
IBW: Ideal Boby Weight
ICU: Intensive Care Unit
IMV: Invasive Mechanical Ventilation
IQR: Interquartile range
NIV: Non-Invasive Ventilation
OR: Odds Ratio
RESP score: Respiratory ECMO survival prediction score
SAPS II: Simplified Acute Physiology Score II
MOFS: Multi Organ Failure Syndrome
VA-ECMO: Veno-Arterial Extracorporeal Membrane Oxygenation
VV-ECMO: Veno-Venous Extracorporeal Membrane Oxygenation

## 1 Introduction

Since the beginning of the Coronavirus (Covid-19) pandemic in 2019, recommendations for the management of critically ill patients have altered both due to greater knowledge of the virus but also to optimize limited ICU resources [1,2]. Veno-venous Extracorporeal Membrane Oxygenation (VV-ECMO) is an efficient ventilatory support which improves the outcome of patients with Acute Respiratory Distress Syndrome (ARDS) refractory to standard therapy. During the pandemic, practitioners were encouraged to transfer the most critically ill patients to large, specialized centers to optimize existing ICU resources [3,4]. Although the VV-ECMO ventilator criteria have not changed for patients with non-Covid-19 ARDS, contraindications, in particular the duration of invasive mechanical ventilation (IMV), are still controversial [[5], [6], [7], [8], [9]]. Initial studies reported increased mortality in patients assisted by VV-ECMO after invasive mechanical ventilation of more than 7 days, while more recent studies show that prolonged IMV was not associated with lower Covid-19-related ARDS survival rates [[10], [11], [12], [13], [14], [15]]. The aim of this study was to investigate if patients with prolonged IMV prior to VV-ECMO (superior to 7 and 10 days) had higher in-hospital mortality than those assisted earlier in refractory Covid-19-related ARDS.

## 2 Materials and methods

1. Study population

This single-center retrospective study included all patients treated with VV-ECMO for refractory Covid-19-related ARDS between January 1, 2020 and May 31, 2022 in the medical and surgical Intensive Care Unit (ICU) of our institution. Some patients were transferred to our center from hospitals which did not have ECMO capability. Exclusion criteria were: age under 18 years at initiation of VV-ECMO, VA-ECMO for circulatory failure and missing data. The study was approved by the Ethics Committee of the French Society of Infectious and Tropical Diseases (IRB 2022/CHU/21). This observational study followed STROBE guidelines.

2. Indications for VV-ECMO.

Our institution is a specialized tertiary care center. Decisions to initiate VV-ECMO are taken during multidisciplinary consultations with intensivists, cardiac surgeons and referring physicians and in accordance with international recommendations on Covid-19 patient management [1,3,4,6,16]. ECMO in COVID-19-related ARDS was used as a last resort and initiated when all other first-line strategies including lung protective ventilation, prone positioning, optimal positive end expiratory pressure (PEEP), or neuromuscular blocking agents had failed. Implantation technique and our management protocol of VV-ECMO are described in appendix 1. VV-ECMO was managed by the intensivist in charge of the patient. Our center adopts a protective ventilation strategy in ARDS patients on ECMO, using a volume-limited controlled ventilation mode pursuing tidal volumes between 2 and 4 ml/kg ideal body weight (IBW), a driving pressure between 5 and 15 cm H2O, and a target plateau pressure of ≤30 cm H2O. VV-ECMO blood and gas flows were adjusted to maintain a peripheral saturation between 90 and 93 %.

3. Periods

Due to waves and the emergence of variants, the study was divided into 4 periods [17]. Period 1, between January 1st, 2020 and January 30th, 2021, corresponds to the first wave on the island during which the original strain (alpha) and B.1.622 (local variant) were predominant. Period 2, between February 1st, 2021 and June 3th, 2021, corresponds to the second wave during which 501Y·V2 (beta variant) was responsible for most hospital admissions and transfers to our tertiary care center [17,18]. Period 3, between July 1st^,^ 2021 and November 30th^,^ 2021, corresponds to the third wave during which B.1.617.2 (delta) was the main circulating strain. Period 4, between December 1st^,^ 2021 and May 31st^,^ 2022, was the fourth wave with B.1.617.2 (delta) and B1.1.529 (omicron) circulating [17,19].

4. Statistical analysis

The primary endpoint was in-hospital mortality. Data was collected using the patient data management system's routine documentation (Crossway®, Cegedim Santé, Paris, France). Categorial variables were expressed as numbers (percent, %) and were compared using the Chi-square test or Fisher's exact test, as appropriate. Quantitative variables were expressed as median and Inter Quartile Range (IQR, represented by 25th-75th percentiles) in non-normally distributed variables. Comparisons between two groups were performed using the Mann-Whitney test. In-hospital mortality risk factors of were identified by comparing pre-VV-ECMO characteristics between survivors and non-survivors and using a backward stepwise logistic regression model. (Only variables with *p*-values ≤0.05 were entered into the logistic regression model). The impact of IMV duration was investigated by comparing patients on VV-ECMO in the 7 days following IMV (Group ≤7 days) with those assisted after 7 days of IMV (Group >7 days). The same comparison was performed between patients assisted in the ten days following IMV (Group ≤10 days) with those assisted after 10 days (Group >10 days). Pre-implantation characteristics and outcomes for each group were compared. Statistical analyses were performed using SPSS statistical software (8.2, Cary, NC, USA).

## 3 Results

1. Descriptive data

During the inclusion period, 64 patients were hospitalized in ICU for Covid-19-related refractory ARDS requiring VV-ECMO. Median age was 52 [44.5; 56.2] years, 42 patients (66 %) were male, median BMI was 29 [25.7; 33.2] kg/m^2^, median SAPS score on admission 34 [24.5; 44] and median RESP-score on implantation 2 [1; 4]). Twenty-one (33 %) were implanted by our mobile ECMO retrieval teams then transferred to our ICU. Twelve (19 %) were transferred from another hospital after implantation by local ICU team.

On ECMO initiation, the median duration of Invasive Mechanical Ventilation (IMV) was 4 [2; 8] days, 60 patients (94 %) were placed in the prone position with a median of 2 [1; 3] sessions. Median driving pressure was 21 [20; 25] cmH2O. Thirty-seven (58 %) were given norepinephrine and five (7.8 %) renal replacement therapy. Study population characteristics and conditions at VV-ECMO initiation are described in Table 1, Table 2. Final blood gases before implantation showed a median P/F ratio of 61 [56; 74], a median PaCO2 of 58 [45; 66] mmHg and a median pH of 7.31 [7.23; 7.41]. Laboratory parameters and blood gases are given in Table 1. Median duration of ECMO was 21 [12; 30] days, total duration of IMV was 33 [24; 53] days and median ICU stay was 40 [27; 58] days.

**Table 1:** Characteristics and laboratory parameters of survivors and non-survivors.

Characteristics	All patients (n = 64)	Survivors group (n = 29)	Non survivors group (n = 35)	*p*
Age, years [IQR]	52 [44.5; 56.2]	50 [41; 54]	53 [47.5; 58]	0.075
Male, n (%)	42 (66)	22 (76)	20 (57)	0.12
BMI, kg/m^2^ [IQR]	29 [25.7; 33.2]	31 [27; 33]	29 [25.2; 34.3]	0.58
Scores				
SAPS II [IQR]	34 [24.5; 44]	33 [24; 42]	34 [25.2; 44]	0.82
RESP Score [IQR]	2 [1; 4]	2 [2; 4]	2 [0.5; 4]	0.2
Comorbidities				
Chronic heart disease, n (%)	5 (7.8)	1 (3.4)	4 (11)	0.37
Chronic lung disease, n (%)	12 (19)	3 (10)	9 (26)	0.12
Chronic renal failure, n (%)	9 (14)	2 (6.9)	7 (20)	0.17
Occlusive arterial disease of limbs, n (%)	1 (1.6)	0 (0)	1 (2.9)	1
Arterial hypertension, n (%)	23 (36)	9 (31)	14 (40)	0.46
Diabetes, n (%)	27 (42)	10 (34)	17 (49)	0.26
Smoking, n (%)	4 (6.2)	2 (6.9)	2 (5.7)	1
Chronic alcoholic use, n (%)	3 (4.7)	1 (3.4)	2 (5.7)	1
Immunosuppression, n (%)	10 (16)	1 (3.4)	9 (26)	**0.017**
Periods				
1st period (Jan 2020–Dec 2020), n (%)	8 (12)	4 (14)	4 (11)	1
2nd period, (Jan 2021–Jun 2021), n (%)	31 (48)	11 (38)	20 (57)	0.13
3rd period, n (Jul 2021–Nov 2021), n (%)	12 (19)	9 (31)	3 (8.6)	**0.022**
4th period (Dec 2021–May 2022), n (%)	13 (20)	5 (17)	8 (23)	0.58
Laboratory values				
Hemoglobin, g/dL [IQR]	10.2 [8.8; 12]	11.3 [9.25; 12.1]	9.3 [8.5; 11.4]	0.069
Platelet count, G/L [IQR]	274 [190; 330]	292 [218; 358]	258 [181; 318]	0.16
Prothrombin Time, % [IQR]	79 [73; 90]	79 [71; 91]	79 [73; 85]	0.99
Fibrinogen, g/L [IQR]	6 [4.8; 8.6]	5.85 [4.59; 9.57]	6.75 [5.2; 8.2]	0.74
Creatinine, μmol/L [IQR]	67 [53.5; 101]	61 [49.5; 96]	68.5 [54.8; 123]	0.33
Blood gases before VV ECMO				
pH, [IQR]	7.31 [7.23; 7.41]	7.34 [7.28; 7.42]	7.28 [7.20; 7.37]	0.12
PaO_2_, mmHg [IQR]	61 [56; 74]	62.5 [55.2; 73.8]	61 [56.5; 74]	0.98
PaO_2_/FiO_2_, mmHg/% [IQR]	61 [56; 74.5]	62.5 [55.8; 73.2]	61 [56.5; 76.5]	0.67
PaCO_2_, mmHg [IQR]	58 [45; 65.5]	58 [45; 63.5]	56 [46; 66]	0.99
SaO_2_,% [IQR]	91 [82; 93]	91 [82; 93]	91 [84; 94]	0.86
Arterial lactate, mmol/L [IQR]	1.6 [1.3; 2.4]	1.8 [1.25; 2.45]	1.55 [1.33; 2.05]	0.74

IQR = Interquartile Range represented by 25th-75th percentiles; n = Number; BMI = Body mass index; SAPS II = simplified acute physiology score II; RESP Score = Respiratory ECMO Survival Prediction Score VV-ECMO: VenoVenous extracorporeal membrane oxygenation. PP = Prone position.

**Table 2:** Conditions before VV-ECMO initiation and outcomes of the survivors and non-survivors groups.

Parameters	All patients (n = 64)	Survivors group (n = 29)	Non-survivors group (n = 35)	*p*
Conditions before VV-ECMO initiation				
Transferred from another hospital on VV-ECMO, n (%)	12 (19)	6 (21)	6 (17)	0.72
Retrieval by Mobile ECMO team, n (%)	21 (33)	10 (34)	11 (31)	0.8
Pulmonary Bacterial infection associated, n (%)	22 (39)	9 (33)	13 (45)	0.38
Pulmonary Embolism associated, n (%)	7 (11)	3 (10)	4 (12)	1
Neuromuscular blocking agent, n (%)	64 (100)	29 (100)	35 (100)	1
Nitric Oxide inhalation, n (%)	35 (57)	17 (59)	18 (56)	0.85
Prone position PP, n (%)	60 (94)	28 (97)	32 (91)	0.62
Number of PP sessions [IQR]	2 [1; 3]	3 [1; 3]	2 [1; 3]	0.18
Compliance, ml/cm H_2_O [IQR]	16 [11; 24]	17 [11; 24]	16 [15; 23]	0.94
Driving Pressure, cmH_2_O [IQR]	21 [20; 25]	20 [16.2; 25.8]	22 [20; 24.5]	0.41
Duration of IMV, days [IQR]	4 [2; 8]	4 [2; 8]	3 [1; 8]	0.27
Norepinéphrine, n (%)	37 (58)	13 (45)	24 (69)	0.056
Doses of norepinephrine >0.1 μg/kg/min, n (%)	20 (35)	5 (19)	15 (50)	**0.013**
Renal Remplacement Therapy n (%)	5 (7.8)	0 (0)	5 (14)	0.058
Size of drainage cannula, French [IQR]	27 [25; 29]	29 [25; 29]	25 [25; 29]	**0.019**
Size of return cannula, French [IQR]	21 [21; 21]	21 [21; 21]	21 [21; 21]	0.7
Duration between:				
1st symptoms – ICUs admission, days [IQR]	8 [6; 9]	7 [5; 9]	9 [7; 12]	**0.016**
1st symptoms – ICUs admission >7 days, n (%)	35 (55)	11 (38)	24 (69)	**0.022**
1st symptoms – VV ECMO, days [IQR]	15 [12; 21]	14 [11; 20]	16 [12; 21]	0.16
1st symptoms - IMV, days [IQR]	10 [8; 13]	9 [7; 13]	11 [9; 14]	**0.016**
ICUs admission - IMV, days [IQR]	2 [0; 5]	1 [0; 5]	2 [0; 5]	0.45
IMV – VV ECMO, days [IQR]	4 [2; 8]	4 [2; 8]	3 [1; 8]	0.27
Prone Position during VV ECMO, n (%)	11 (17)	6 (21)	5 (14)	0.53
Number of PP sessions [IQR]	1 [1,2]	2 [1,3]	1 [1,1]	0.78
Outcomes				
In-hospital mortality, n (%)	35 (55)	0 (0)	35 (100)	**< 0.001**
ICU stay, days [IQR]	40 [27; 58]	50 [38; 58]	30 [22; 44]	**<0.01**
Duration of VV ECMO, days [IQR]	21 [12; 30]	19 [14; 25]	24 [10; 33]	0.58
Duration of total IMV, days [IQR]	33 [24; 53]	41 [29; 59]	28 [19; 45]	**0.019**

IQR = Interquartile Range represented by 25th-75th percentiles; VV-ECMO: VenoVenous extracorporeal membrane oxygenation. PP = Prone position, IMV: Invasive Mechanical Ventilation; ICU: Intensive Care Unit.

**Fig. 1:**
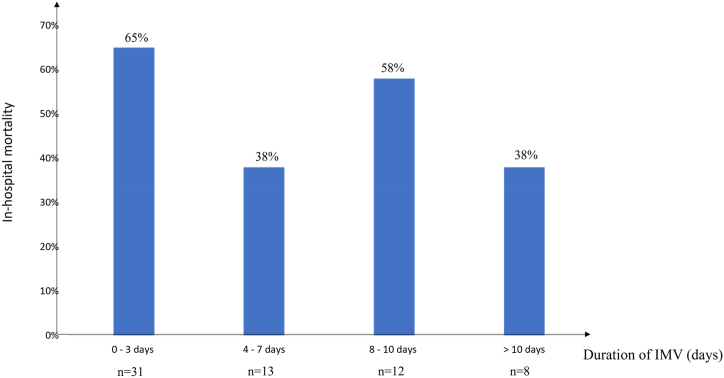
In-hospital mortality vs. duration of invasive mechanical ventilation (IMV) prior to VV-ECMO initiation. n corresponding to the number of patients assisted for each duration of invasive mechanical ventilation (days).

In-hospital mortality was 55 %. In-hospital mortality as a function of IMV duration is shown in Fig. 1. Main causes of death were septic shock (45 %) and hemorrhagic complications (20 %). Eleven patients (17 %) were put in the prone position during VV-ECMO with a median of 1 [1,2] sessions. Differences between survivors and non-survivors are described in Table 1, Table 2 Variables used in the backward stepwise logistic regression model were immunosuppression, norepinephrine >0.1 μg/kg/min, size of drainage cannula and duration of initial symptoms – ICU admission >7 days. One independent pre-implantation risk factor for mortality was identified in multivariate analysis (Table 3) as norepinephrine >0.1 μg/kg/min (Odds ratio = 8.4 [95 % CI 1.5–47], p = 0.016).

2. Comparison between the Group ≤7 days and the Group >7 days.

**Table 3:** Multivariate analysis of pre-implantation mortality risk factors.

Variables	Odds ratio [95 % CI]	*p*
Norepinephrine >0.1 μg/kg/min	8.4 [1.5–47]	0.016
Size of drainage cannula	0.7 [0.5–1]	0.077
Duration 1st symptoms – ICU	4.6 [0.9–22]	0.055
Immunosuppression	11.1 [0.9–137]	0.062

CI: confidence Interval, ICU:Intensive Care Unit.

**Fig. 2:**
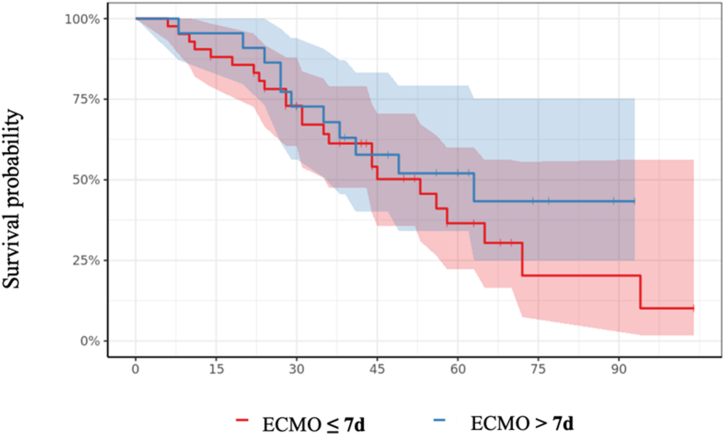
Survival probabilities plotted for all patient groups as a function of pre-ECMO IMV duration ≤7 and >7 days.

We compared 42 patients on VV-ECMO with a pre-ECMO IMV duration of ≤7 days with 22 patients initiated after 7 days of IMV. Both groups had similar demographics and medical history, except for heart disease, which was greater in the Group >7 days group (*p* = 0.044). SAPS scores were similar but RESP scores were lower in the Group >7 days with a significantly longer median duration of invasive ventilation: 10 [8; 11] days vs. 2 [1; 4] (*p* < 0.001). On the day of cannulation, patients implanted after 7 days of IMV were more likely to be treated for pulmonary bacterial infection (p < 0.01), had received more sessions in prone position (p < 0.001) and compliance and driving pressure were more impaired in the >7 days group (=0.05 and *p* < 0.01 respectively). The last biology report before cannulation in the > 7-day group showed more severe anemia and higher fibrinogen, PaCO2 was significantly higher but with no significant acidosis in the Group >7 days. Hypoxaemia was comparable between the groups. No excess in-hospital mortality was observed in patients cannulated after 7 days of IMV compared to those assisted earlier (50 % vs. 57 %, p = 0.59, Fig. 2). Demographic data and detailed baseline information at ECMO initiation is given in Table 4, Table 5.

3. Comparison between Group ≤10 days and Group >10 days.

**Table 4:** Characteristics and laboratory parameters of Group ≤7 days and Group >7 days.

Characteristics	All patients (n = 64)	Group ≤7 days (n = 42)	Group >7 days (n = 22)	*p*
Age, years [IQR]	52 [44.5; 56.2]	51 [45; 56.8]	52 [44.2; 56]	1
Male, n (%)	42 (66)	26 (62)	16 (73)	0.39
BMI, kg/m^2^ [IQR]	29 [25.7; 33.2]	30 [26.2; 33]	27.5 [24.2; 33.9]	0.23
Scores				
SAPS II [IQR]	34 [24.5; 44]	37 [25.2; 48.2]	29 [24; 35]	0.08
RESP Score [IQR]	2 [1; 4]	4 [2; 5]	1.5 [0.25; 2]	**< 0.001**
Comorbidities				
Chronic heart disease, n (%)	5 (7.8)	1 (2.4)	4 (18)	**0.044**
Chronic lung disease, n (%)	12 (19)	8 (19)	4 (18)	1
Chronic renal failure, n (%)	9 (14)	7 (17)	2 (9.1)	0.27
Occlusive arterial disease of the lower limbs, n (%)	1 (1.6)	1 (2.4)	0 (0)	1
Arterial hypertension, n (%)	23 (36)	17 (40)	6 (27)	0.3
Diabetes, n (%)	27 (42)	19 (45)	8 (36)	0.49
Smoking, n (%)	4 (6.2)	2 (4.8)	2 (9.1)	0.6
Chronic alcoholic use, n (%)	3 (4.7)	1 (2.4)	2 (9.1)	0.27
Immunosuppression, n (%)	10 (16)	8 (19)	2 (9.1)	0.47
Periods				
1st period (Jan 2020–Dec 2020), n (%)	8 (12)	6 (14)	2 (9.1)	0.7
2nd period, (Jan 2021–Jun 2021), n (%)	31 (48)	20 (48)	11 (50)	0.86
3rd period, n (Jul 2021–Nov 2021), n (%)	12 (19)	9 (21)	3 (14)	0.52
4th period (Dec 2021–May 2022), n (%)	13 (20)	7 (17)	6 (27)	0.34
Laboratory values				
Hemoglobin, g/dL [IQR]	10.2 [8.8; 12]	11.2 [9.3; 12.2]	9.1 [8.5; 11]	**0.02**
Platelet count, G/L [IQR]	274 [190; 330]	274 [200; 328]	260 [184; 351]	0.69
Prothrombin Time, % [IQR]	79 [73; 90]	77 [72; 89]	81 [73; 91]	0.67
Fibrinogen, g/L [IQR]	6 [4.8; 8.6]	5.2 [4; 8]	8 [5.6; 11.4]	**0.017**
Creatinine, μmol/L [IQR]	67 [53.5; 101]	70 [54.5; 109]	60.5 [45.8; 86.2]	0.19
Blood gases before VV ECMO				
pH, [IQR]	7.31 [7.23; 7.41]	7.33 [7.26; 7.41]	7.29 [7.20; 7.35]	0.35
PaO_2_, mmHg [IQR]	61 [56; 74]	62 [57; 74.5]	60.5 [56; 73.5]	0.35
PaO_2_/FiO_2_, mmHg/% [IQR]	61 [56; 74.5]	63 [60; 75]	59.5 [53.8; 73.5]	0.19
PaCO_2_, mmHg [IQR]	58 [45; 65.5]	53 [44; 64]	60.5 [56; 73.5]	**0.018**
SaO_2_,% [IQR]	91 [82; 93]	91 [86; 94]	89 [82; 93]	0.29
Arterial lactate, mmol/L [IQR]	1.6 [1.3; 2.4]	1.75 [1.30; 2.55]	1.60 [1.30; 2.10]	0.65

IQR = Interquartile Range represented by 25th-75th percentiles; n = Number; BMI = Body mass index; SAPS II = simplified acute physiology score II; RESP Score = Respiratory ECMO Survival Prediction Score VV-ECMO: VenoVenous extracorporeal membrane oxygenation. PP = Prone position.

**Table 5:** Conditions before VV-ECMO initiation and outcomes of Group ≤7 days and Group >7 days.

Characteristics	All patients (n = 64)	Group ≤7 days (n = 42)	Group >7 days (n = 22)	*p*
Characteristics at VV-ECMO initiation				
Transferred from another hospital on VV-ECMO, n (%)	12 (19)	6 (14)	6 (27)	0.31
Retrieval by Mobile ECMO team, n (%)	21 (33)	15 (36)	6 (27)	0.49
Pulmonary Bacterial infection associated, n (%)	22 (39)	9 (26)	13 (62)	**< 0.01**
Pulmonary Embolism associated, n (%)	7 (11)	3 (7.1)	2 (9.1)	1
Neuromuscular blocking agent, n (%)	64 (100)	42 (100)	22 (100)	1
Nitric Oxide inhalation, n (%)	35 (57)	20 (51)	15 (68)	0.20
Prone position PP, n (%)	60 (94)	38 (90)	22 (100)	0.29
Number of PP sessions [IQR]	2 [1; 3]	1 [1; 3]	3 [2; 6]	**< 0.001**
Compliance, ml/cm H_2_O [IQR]	16 [11; 24]	19 [14; 26]	14 [10; 17]	**0.05**
Driving Pressure, cmH_2_O [IQR]	21 [20; 25]	20 [14; 23]	24.5 [21; 29.8]	**< 0.01**
Duration of IMV, days [IQR]	4 [2; 8]	2 [1; 4]	10 [8; 11]	**< 0.001**
Norepinéphrine n (%)	37 (58)	27 (64)	10 (45)	0.15
Doses of norepinephrine >0.1 μg/kg/min, n (%)	20 (35)	13 (36)	7 (33]	0.83
Renal Remplacement Therapy n (%)	5 (7.8)	3 (7.1)	2 (9.1)	1
Size of drainage cannula, French [IQR]	27 [25; 29]	25 [25; 29]	29 [25; 29]	0.066
Size of return cannula, French [IQR]	21 [21; 21]	21 [20; 21]	21 [21; 21]	0.052
Duration between:				
1st symptoms – ICUs admission, days [IQR]	8 [6; 9]	8 [5; 10]	8 [7; 9]	0.79
1st symptoms – ICUs admission >7 days, n (%)	35 (55)	23 (55)	12 (55)	1
1st symptoms – VV ECMO, days [IQR]	15 [12; 21]	13 [11; 16]	20 [16; 22]	**< 0.001**
1st symptoms - IMV, days [IQR]	10 [8; 13]	11 [8; 15]	9 [7; 13]	0.20
ICUs admission - IMV, days [IQR]	2 [0; 5]	3 [0; 7]	1 [0; 3]	0.063
IMV – VV ECMO, days [IQR]	4 [2; 8]	2 [1; 4]	10 [8; 11]	**< 0.001**
Prone Position during VV ECMO, n (%)	11 (17)	3 (14)	8 (19)	0.73
Number of PP sessions [IQR]	1 [1,2]	1 [1,2]]	1 [1,2]	0.65
Outcomes				
In-hospital mortality, n (%)	35 (55)	24 (57)	11 (50)	0.59
ICU stay, days [IQR]	40 [27; 58]	37 [24; 53]	44 [29; 63]	0.18
Duration of VV ECMO, days [IQR]	21 [12; 30]	21 [9; 31]	23 [15; 27]	0.57
Duration of total IMV, days [IQR]	33 [24; 53]	30 [20; 46]	40 [28; 61]	0.056

IQR = Interquartile Range represented by 25th-75th percentiles; VV-ECMO: VenoVenous extracorporeal membrane oxygenation. PP = Prone position, IMV: Invasive Mechanical Ventilation; ICU: Intensive Care Unit.

**Fig. 3:**
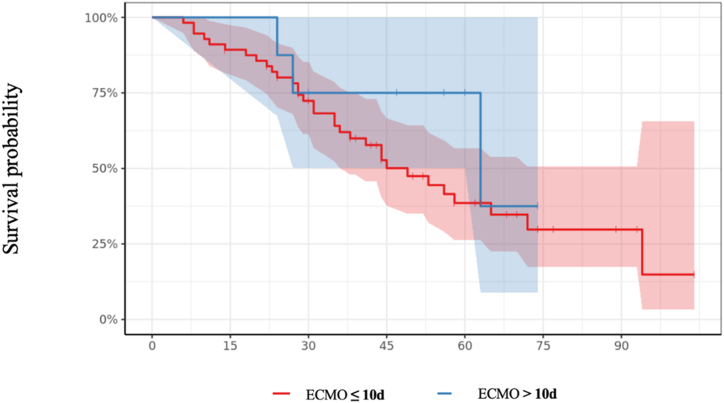
Survival probabilities plotted for all patient groups as a function of pre-ECMO IMV duration ≤10 and >10 days.

Fifty-six patients were implanted for up to 10 days with a median IMV duration of 3 days [2; 7]. They were compared to the group of 8 patients cannulated after the 10th day of IMV, with a median IMV duration of 13 days [11; 15] (p < 0.001). Both groups had similar comorbidities but the Group >10 days had a significant difference in the number of PP sessions (p = 0.02) and a higher DP (p = 0.017). Demographic data and detailed baseline information at ECMO initiation is given in Table 6, Table 7. No excess in-hospital mortality was observed between the two groups (38 % for the Group >10 days group vs. 57 % for the Group ≤10 days, *p* = 0.45, Fig. 3).

**Table 6:** Characteristics and laboratory parameters of Group ≤10 days and Group >10 days.

Characteristics	All patients (n = 64)	Group ≤10 days (n = 56)	Group >10 days (n = 8)	*p*
Age, years [IQR]	52 [44.5; 56.2]	52 [45; 56.2]	52 [38; 54.8]	0.58
Male, n (%)	42 (66)	35 (62)	7 (88)	0.25
BMI, kg/m^2^ [IQR]	29 [25.7; 33.2]	30 [25.9; 34]	26.8 [24; 29.8]	0.21
Scores				
SAPS II [IQR]	34 [24.5; 44]	35 [24.5; 45]	28 [24.8; 30]	0.15
RESP Score [IQR]	2 [1; 4]	3 [1; 4]	1.5 [1; 2]	0.051
Comorbidities				
Chronic heart disease, n (%)	5 (7.8)	5 (8.9)	0 (0)	1
Chronic lung disease, n (%)	12 (19)	12 (21)	0 (0)	0.33
Chronic renal failure, n (%)	9 (14)	9 (16)	0 (0)	0.59
Occlusive arterial disease of the lower limbs, n (%)	1 (1.6)	1 (1.8)	0 (0)	1
Arterial hypertension, n (%)	23 (36)	22 (39)	1 (12)	0.24
Diabetes, n (%)	27 (42)	26 (46)	1 (12)	0.12
Smoking, n (%)	4 (6.2)	3 (5.4)	1 (12)	0.42
Chronic alcoholic use, n (%)	3 (4.7)	2 (3.6)	1 (12)	0.33
Immunosuppression, n (%)	10 (16)	10 (18)	0 (0)	0.34
Periods				
1st period (Jan 2020–Dec 2021), n (%)	8 (12)	8 (14)	0 (0)	0.58
2nd period, (Jan 2021–Jun 2021), n (%)	31 (48)	27 (48)	4 (50)	1
3rd period, n (Jul 2021–Nov 2021), n (%)	12 (19)	10 (18)	2 (25)	0.64
4th period (Dec 2021–May 2022), n (%)	13 (20)	11 (20)	2 (25)	0.66
Laboratory values				
Hemoglobin, g/dL [IQR]	10.2 [8.8; 12]	10.4 [8.8; 12]	9.2 [8.75; 10.7]	0.35
Platelet count, G/L [IQR]	274 [190; 330]	273 [190; 330]	284 [180; 326]	0.9
Prothrombin Time, % [IQR]	79 [73; 90]	80 [73; 90]	74 [72; 90]	0.84
Fibrinogen, g/L [IQR]	6 [4.8; 8.6]	5.7 [4.50; 8.45]	7.20 [6; 11.1]	0.12
Creatinine, μmol/L [IQR]	67 [53.5; 101]	67.5 [53.8; 108]	61 [51.5; 89.5]	0.79
Blood gases before VV ECMO				
pH, [IQR]	7.31 [7.23; 7.41]	7.32 [7.24; 7.41]	7.26 [7.20; 7.33]	0.29
PaO_2_, mmHg [IQR]	61 [56; 74]	61 [56; 74]	68 [55.5; 75]	0.84
PaO_2_/FiO_2_, mmHg/% [IQR]	61 [56; 74.5]	61 [56; 74]	68 [55.5; 78.5]	0.6
PaCO_2_, mmHg [IQR]	58 [45; 65.5]	55 [45; 64]	69 [55; 81]	0.074
SaO_2_,% [IQR]	91 [82; 93]	91 [84; 93]	91 [79; 93]	0.81
Arterial lactate, mmol/L [IQR]	1.6 [1.3; 2.4]	1.70 [1.33; 2.65]	1 [0.95; 1.75]	0.063

IQR = Interquartile Range represented by 25th-75th percentiles; n = Number; BMI = Body mass index; SAPS II = simplified acute physiology score II; RESP Score = Respiratory ECMO Survival Prediction Score VV-ECMO: VenoVenous extracorporeal membrane oxygenation. PP = Prone position.

**Table 7:** Conditions before VV-ECMO initiation and outcomes of Group ≤10 days and Group >10 days.

Characteristics	All patients (n = 64)	Group ≤10 days (n = 56)	Group >10 days (n = 8)	*p*
Characteristics at VV-ECMO initiation				
Transferred from another hospital on VV ECMO, n (%)	12 (19)	10 (18)	2 (25)	0.64
Retrieval by Mobile ECMO team, n (%)	21 (33)	18 (32)	3 (38)	1
Pulmonary Bacterial infection associated, n (%)	22 (39)	17 (35)	5 (71)	0.099
Pulmonary Embolism associated, n (%)	7 (11)	5 (9.1)	2 (25)	0.21
Neuromuscular blocking agent, n (%)	64 (100)	56 (100)	8 (100)	1
Nitric Oxide inhalation, n (%)	35 (57)	30 (57)	5 (62)	1
Prone position PP, n (%)	60 (94)	52 (93)	8 (100)	1
Number of PP sessions, cmH_2_O [IQR]	2 [1; 3]	2 [1; 3]	4 [3; 6]	**0.02**
Compliance, ml/cm H_2_O [IQR]	16 [11; 24]	17 [12; 26]	13 [10; 16]	0.14
Driving Pressure, cmH_2_O [IQR]	21 [20; 25]	20 [20; 24]	29 [26; 30]	**0.017**
Duration of IMV, days [IQR]	4 [2; 8]	3 [1; 7]	13.5 [11; 15]	**< 0.001**
Norepinéphrine n (%)	37 (58)	34 (61)	3 (38)	0.27
Doses of norepinephrine >0.1 μg/kg/min, n (%)	20 [35]	19 [39]	1 [12]	0.24
Renal Remplacement Therapy n (%)	5 (7.8)	4 (7.1)	1 (12)	0.5
Size of drainage cannula, French [IQR]	27 [25; 29]	27 [25; 29]	29 [26; 29]	0.4
Size of return cannula, French [IQR]	21 [21; 21]	21 [21; 21]	21 [21; 21]	0.96
Duration between:				
1st symptoms – ICUs admission, days [IQR]	8 [6; 9]	8 [5; 9]	9 [7; 10]	0.46
1st symptoms – ICUs admission >7 days, n (%)	35 (55)	30 (54)	5 (63)	0.72
1st symptoms – VV ECMO, days [IQR]	15 [12; 21]	15 [12; 18]	22 [21; 25]	**<0.001**
1st symptoms - IMV, days [IQR]	10 [8; 13]	10 [8; 13]	10 [9; 12]	0.89
ICUs admission - IMV, days [IQR]	2 [0; 5]	2 [0; 5]	0 [0; 1]	0.1
IMV – VV ECMO, days [IQR]	4 [2; 8]	15 [12; 18]	22 [21; 25]	**<0.01**
Prone Position during VV ECMO, n (%)	11 (17)	9 (16)	2 [25]	0.62
Number of PP sessions [IQR]	1 [1,2]	2 [1,2]	1 [1,2]	0.85
Outcomes				
In-hospital mortality, n (%)	35 (55)	32 (57)	3 (38)	0.45
ICU stay, days [IQR]	40 [27; 58]	39 [26; 57]	51 [29; 61]	0.39
Duration of VV ECMO, days [IQR]	21 [12; 30]	21 [12; 30]	22 [14; 25]	0.86
Duration of total IMV, days [IQR]	33 [24; 53]	32 [22; 49]	48 [28; 60]	0.24

IQR = Interquartile Range represented by 25th-75th percentiles; VV-ECMO: VenoVenous extracorporeal membrane oxygenation. PP = Prone position, IMV: Invasive Mechanical Ventilation; ICU: Intensive Care Unit.

## 4 Discussion

In our study, patients with refractory Covid-19-related ARDS assisted by VV-ECMO after prolonged IMV had no higher in-hospital mortality than those assisted earlier. In-hospital mortality was 55 %, which may appear higher than previously reported in patients treated with VV-ECMO during the first wave of the Covid-19 pandemic but was similar to that reported in larger cohorts in subsequent waves [7]. Lebreton et al. reported a 90-day mortality of 54 % in patients on VV-ECMO and Barbaro et al. reported an in-hospital mortality of between 37 % and 59 % [10,11,13]. In a nationwide cohort of 429 patients, Nesseler et al. reported an in-hospital mortality of 49 % [9]. In more selected Covid-19 patient cohorts due to resource constraints during the pandemic, some authors reported in-hospital mortality at between 35 and 39 % [12,20]. However, these studies were conducted before the emergence of variants, notably 501Y·V2 (beta) and B.1.617.2 (delta) strains which are associated with poorer outcomes and increased mortality. Puech et al. reported increased mortality in patients infected with the beta strain compared to patients infected with the original strain [18,21]. Twohig et al. described higher hospital admission rates in patients infected with the delta strain and Schmidt et al. found elevated virulence and poorer outcomes with the delta strain in patients on ECMO for Covid-ARDS [22,23]. Sixty-seven percent of our patients (n = 43) were hospitalized in periods 2 and 3, during which the beta and delta strains were predominant, which may explain the in-hospital mortality observed in our study.

In our population, patients placed on VV-ECMO after >7 days (and 10 days) of IMV had the same outcomes as those placed on VV-ECMO earlier. Initial studies showed higher mortality in patients on VV-ECMO with prolonged IMV. Lebreton et al. found that shorter intervals between intubation and ECMO (odds ratio 0.91 per day decrease) was associated with improved 90-day survival [10]. In a cohort of 1215 patients, Lorusso et al. reported worse outcomes in Covid-19 patients on ECMO for cannulation ≥4 days and in another emulated target trial, ECMO initiated within four days of IMV was associated with a lower mortality [13,24]. Nesseler et al. observed an increased mortality for patients on ECMO for more than 7 days compared to durations of less than 2 days [9]. Owing to these results and data pertaining to patients cannulated before 2012 reporting a strong correlation between mortality and IMV duration, initial guidelines did not recommend the use of ECMO in patients on IMV for more than 7 days, particularly in the context of the pandemic and resource limitations [4,5]. During the Covid-19 epidemic in our region, the reorganization of care and the creation of additional hospital space and critical care beds made it possible to care for all patients. It was decided not to restrict use of VV-ECMO in patients presenting with few comorbidities or factors associated with a good prognosis. Furthermore, even if the differences were not significant, patients assisted after a prolonged IMV were more selected and presented with fewer co-morbidities. In the Group >10 days, no patient had chronic heart, renal or pulmonary disease; this may have improved their prognosis. Conversely, more patients in the Group >7 days presented with chronic heart disease and pulmonary bacterial infections.

In our cohort, patients placed on VV-ECMO after 7 days (or 10 days) of IMV presented equivalent in-hospital mortality than those assisted earlier (50 % versus 57 %, *p* = 0.59, and 38 % versus 57 %, *p* = 0.45, respectively). In a cohort of 94 patients in Chile, Diaz et al. indicated that IMV duration prior to ECMO had no impact on survival [20]. In another cohort of 319 patients at 24 centers, IMV duration prior to ECMO was not associated with increased mortality. Olivier et al. found similar results in a cohort of 56 patients and concluded that IMV duration should not be taken into account when considering ECMO [14,25]. Finally, Hermann et al. reported that in a cohort of 101 Covid-19 patients on ECMO, IMV duration was not associated with increased mortality and proposed an individual approach using the RESP score to balance the risks and benefits of ECMO [15]. These new data have led to the recommendation to not take into account IMV duration as a primary determinant for ECMO candidacy [8]. In our study, the RESP score did not differ significantly between survivors and non-survivors, which did not allow this single score to determine outcomes and prognosis in our population.

Patients presented with significant alterations in pulmonary compliance (median compliance 16 [11; 24] ml/cm H_2_O) and driving pressure (medial DP 21 [20; 25] cm H_2_O). There was no significant difference in these figures between survivors and non-survivors. However, these alterations became more pronounced the later the patients were assisted, and the chronological evolution of these parameters mirrored the progression of Covid-19-related pneumonia. Studies highlight different ventilatory profiles and show that the alteration of compliance or driving pressure varies greatly from one population to another. Thus, in Hermann's study, patients presented with a moderate alteration of driving pressure (mean DP = 14 ± 2 cm H_2_O) at ECMO initiation [15]. In the study by Lebreton and Nessler, driving pressures were respectively 18 [[14], [15], [16], [17], [18], [19], [20], [21]] and 17 [[14], [15], [16], [17], [18], [19], [20]] cm H_2_O. Our cohort showed similar pulmonary compliance to the study by Olivier et al. (median compliance 20 [[15], [16], [17], [18], [19], [20], [21], [22], [23]] ml/cm H_2_O).

Multiple Covid-19 phenotypes have been reported and two different patterns can be described in patients with respiratory failure [16,26]. Type L appeared at the beginning of the pandemic. Patients presented with high compliance, low ventilation-to-perfusion (VA/Q) ratio, low lung weight and recruitabiliby. This pattern was responsible for “Happy Hypoxia” described at the beginning of pandemic [26,27]. Appearing later, type H was the consequence of injury caused by high stress ventilation and was associated with physiopathological changes found in conventional ARDS, low compliance, high right to left shunt and high lung weight which better corresponds to the ventilatory characteristics of our patient population. The evolution of each pattern depended on host response, physiological reserves and comorbidities. For each pattern, different ventilation management were proposed [26]. Thus, different phenotypes were not described in the study and changing recommendations, i.e. airway management or use of corticosteroids (which can improve the prognosis of many patients), may have contradicted results on the impact of IMV duration [1,7,28,29]. Lockdowns caused a delay in the arrival of the epidemic to our island and the vast majority of patients admitted to intensive care benefited from corticosteroid therapy and initial HFNO ventilation support [17,18].

Our study had limitations. Firstly, it is a single-center study and our results cannot be extrapolated to centers with different populations and constraints. Secondly, duration of non-invasive ventilation (NIV) or HFNO was not recorded. It is possible that patients had early or delayed intubation due to changes in airway management recommendations. However, due to the late arrival of the epidemic in our island, our practices remained unchanged throughout the duration of the study. Patients were generally admitted to ICU following failure of conventional oxygen therapy. HFNO was preferred to NIV which was still used respecting the usual recommendations [1,2]. Finally, our study is based on a small cohort and the data needs to be confirmed on a larger population. The only risk factor associated with mortality found in our study is norepinephrine >0.1 μg/kg/min (OR: 8.4 [95 % IC 1.5–47]; *p* = 0.016). The presence of circulatory or other organ failure is typically associated with poorer outcomes [9,12,13]. However, as our statistical data was based on a small cohort, this necessarily resulted in a limited choice of variables in the determination of mortality risk factors. This made it impossible to determine all possible risk factors.

## 5 Conclusion

This study suggested that patients assisted after prolonged IMV had the same prognosis to those assisted earlier in refractory Covid-19-related ARDS requiring VV-ECMO respiratory support. Therefore, prolonged ventilation of more than 7–10 days should not contraindicate VV-ECMO support. An individual approach is necessary to balance the risks and benefits of ECMO in this population.

## Formatting of funding sources

This research did not receive any specific grant from funding agencies in the public, commercial or not-for-profit sectors.

## Human or animals rights

Not Applicable.

## Informed consent and patient details

The authors declare that this report does not contain any personal information that could lead to the identification of the patient(s).

## Disclosure of interest

The authors declare that they have no known competing financial or personal relationships that could be viewed as influencing the work reported in this paper.

## Funding

This work did not receive any grant from funding agencies in the public, commercial, or not-for-profit sectors.

## Data availability statement

Data associated with the study have not been deposited into a publicly available repository and will be made available on request.

## CRediT authorship contribution statement

**Charles Vidal:** Writing – review & editing, Writing – original draft, Supervision, Investigation, Conceptualization. **Mathilde Nativel:** Writing – original draft, Investigation, Data curation. **Puech Bérénice:** Investigation. **Poirson Florent:** Validation. **Cally Radj:** Validation. **Laurence Dangers:** Validation. **Braunberger Eric:** Validation. **Julien Jabot:** Validation. **Nicolas Allou:** Validation, Methodology, Conceptualization. **Jérôme Allyn:** Validation, Methodology, Formal analysis.

## Declaration of competing interest

The authors declare that they have no known competing financial interests or personal relationships that could have appeared to influence the work reported in this paper.
