# Supplementary material for: Duration of Invasive Mechanical Ventilation before Veno-Venous ExtraCorporeal Membrane Oxygenation for Covid-19 related Acute Respiratory Distress Syndrome: The experience of a tertiary care center
Source: Heliyon. 2024 May 29;10(11):e31811. doi: 10.1016/j.heliyon.2024.e31811 (PMC11176752; doi:10.1016/j.heliyon.2024.e31811)
Supplement: Multimedia component 1 [file mmc1.pdf]

Comité d'Ethique de Recherche en Maladies Infectieuses et Tropicales

Institutional Review Board N° IRB00011642

Maison de l'Infectiologie
21 rue Beaurepaire
75010 PARIS

Président
Pr Vincent LE MOING

Secrétaire
Mme Nathalie GASTELLIER

Membres :
Pr Louis BERNARD
Pr Diane DESCAMPS
Pr Vincent DUBEE
Dr Aurélia EDEN
Pr Marie KEMPF
Dr Jean-Philippe LANOIX
Pr Vincent LE MOING
Pr Gilles PIALOUX
Dr Valérie POURCHER
Pr Pierre TATTEVIN

Paris, le 12/07/2023

**N° CER-MIT**	2022-0907-2
**Titre de la recherche**	Late venovenous Extracorporeal Membrane Oxygenation in patients with refractory acute respiratory distress syndrome due to SARS-CoV-2 / Impact sur la mortalité de la ventilation mécanique tardive chez les patients assistés tardivement en ECMO veino veineuse – Etude LEMOC
**Responsable scientifique**	Dr. Mathilde Nativel
**Responsable de traitement**	CHU Nord de La Réunion

Le 10/07/2023, le projet susmentionné a été revu par le CER-MIT après soumission d'une révision du projet initial qui avait obtenu l'avis réservé du Comité le 19 Septembre 2022. Les documents suivants ont été joints pour l'examen du dossier :

- Protocole : Version 2 du 30/04/2023
- Formulaire de soumission CER-MIT dument rempli
- Courrier d'information : Version 2 du 30/04/2023

Après évaluation des documents de l'étude, le CER-MIT a émis l'avis suivant :

AVIS FAVORABLE

En effet, au regard des documents évalués, le CER-MIT considère que du point de vue :

- **Réglementaire :** Le protocole n'implique pas la personne humaine comme défini dans le code de la santé publique (Code de la Santé Publique - Article R1121-1) et ne nécessite donc pas l'avis délibératif d'un Comité de Protection Personnes et/ou de l'autorité de santé compétente ;
- **Rationnel et intérêt scientifique et médical :** Le rationnel de l'étude est justifié, l'étude permettra d'apporter des données intéressantes à la communauté médicale.
- **Ethique :** Le comité n'a pas noté de problématique d'éthique sur le recueil des données.

Le CER-MIT rappelle qu'il n'est pas compétent au regard de la loi Informatiques et Libertés et du Règlement Général sur la Protections des données. Il est à la charge du responsable de traitement de s'y mettre en conformité.

Le comité rappelle qu'il incombe au responsable scientifique de s'assurer que tous les collaborateurs associés à ce projet sont informés des conditions d'approbation et des documents approuvés.

Le comité rappelle que le responsable de l'étude est tenu de fourni au CER-MIT **un rapport final de l'étude ou la publication approuvée**.

Bien cordialement,

Pr Vincent LE MOING
Président du CER-MIT
