# Supplementary material for: Duration of Invasive Mechanical Ventilation before Veno-Venous ExtraCorporeal Membrane Oxygenation for Covid-19 related Acute Respiratory Distress Syndrome: The experience of a tertiary care center
Source: Heliyon. 2024 May 29;10(11):e31811. doi: 10.1016/j.heliyon.2024.e31811 (PMC11176752; doi:10.1016/j.heliyon.2024.e31811)
Supplement: Multimedia component 3 [file mmc3.docx]

EQUIPMENT (depending on availability)

Before assembly, always ask the perfusionists to install 2 taps on the venous side for Renal Replacement Therapy (RRT).

- CPA (cardio pulmonary arrest): Sorin circuit
- Rescue ECMO (backup) => Sorin circuit
- Post cardiotomy indication: Sorin circuit
- ARDS - acute respiratory distress syndrome: Rotaflow (Maquet)
- MCAU (Mobile Circulatory Assistance Unit + Medical Evacuation: Cardiohelp (Maquet)

If prolonged resuscitation is necessary for more than 15 days, change from Sorin to Maquet circuits (Rotaflow or Cardiohelp).

**CHANGING CIRCUITS**

Sorin (certified for 7 days) => change on day 14 at the latest

Maquet (certified for 21 days) => change on day 21 at the latest

**VV (VenoVenous) ECMO FOR ARDS (Acute Respiratory Distress Syndrome)**

**IMPLANTATION:**

Firstly:

- Femoral-right internal jugular (reinjection)
- Or femoro-femoral approach
- Right internal jugular using double current cannula (Eg. AVALON®)
- Pediatrics: < 25-30kg: jugulo-femoral approach (reinjection)
- If possible, place central catheter under ultrasound guidance into the right internal jugular (verified with bedside X-ray) change under guidance for cannula implantation.

UFH bolus (unfractionated heparin) 50 -100 IU / kg before starting ECMO

Initial setting: flow rate 4l/min (50 to 100 ml/Kg/min). Flow rate 4l/min subsequently adjusting to PaCO2, FiO² 100% (Reminder: flow rate is the main determinant of oxygenation level).

Immediately check the position of the cannulas by x-ray and/or TTE (Transthoracic Echocardiography) and/or TEE (Transesophageal Echocardiography) before unclamping.

Positioning mark:

• Drainage cannula at the LVC – RA junction (lower vena cava – right atrium)

• Reinjection cannula in the upper vena cava sector

**MANAGEMENT**

Oxygenation: OBJECTIVE: SpO² > 90-93%. It can sometimes be difficult to obtain despite an increase in flow from recirculation. PaCO² correction: Rapid elimination of CO² by fresh gas flow provided that the minimum ECMO flow is 10-15 ml/kg/min.

**How to optimize oxygenation:**

1. Recirculation can be a major problem

• Risk factors are insufficient distance between the 2 cannulas, hypovolemia, low cardiac output, excessive ECMO flow.

• Reduce recirculation = cannula position, filling, flow adaptation

2. Increase ECMO flow (flow = main factor in oxygenation): 5–6 l/min or 3 l/m² or > 60-70% of cardiac output (requires large drainage cannula).

• Adults: Typically 60 ml/kg/min

• Children: 90 ml/kg;

- Infants: 120 ml/kg

3. Transfusion for Haemoglobin (Hb) > 8-10 g/dl

4. Reduce oxygen consumption = sedation (with or without curarization).

5. Reduce cardiac output

=> in cases of unsuitable sinus tachycardia - Esmolol Intravenous Electric Syringe

**Mechanical ventilation:**

• Ultra protective ventilation: CAV (controlled assisted ventilation) - Vt (tidal volume) 2-4 ml/kg (or Insufflation Pressure at 25cmH20), FiO² as low as possible, PEEP 5-15cm H²O (be aware of venous return and hemodynamics), Fr 4-6/min, prolonged I:E. Pplateau < 30cmH20

**Anticoagulation:**

Reminder: Preheparinated circuit

Long term anticoagulation for the circuits

TCA: 2 to 3 times Control or Antixa

UFH 0.2 to 0.4 IU/ML

Asprin?

In cases where anticoagulation is contraindicated:

ECMO VV: possible in the long run without anticoagulation but monitor the formation of thrombi in the circuit, appearance of CIVD (indicates a macroscopically invisible thrombus or thrombus at the end of the cannulas). Shorter circuit lifespan.

**Fluid Balance:**

- Dry aspect
- Diuretics as soon as possible

**Specific Monitoring :**

- No specific biological surveillance apart from blood gases, hemoglobin, and antixa.
- Exercise vigilance if ECMO does not use anti-coagulation
  - Thrombus in the oxygenation membrane

**Withdrawal from VV ECMO:**

- No minimum or maximum compulsory resuscitation time
- Healing of pulmonary pathology with progressive reintroduction of conventional assisted or controlled ventilation Vt 6 ml / kg, Pplateau <30 cm H²O, PEEP 5-12 cm H²O, FIO² <60%
- Stopping the flow (fresh gas flow = 0) / maintenance of ECMO blood flow
- If clinical signs and gas are stable after 1 to 4 h = decanulation
- Decanulation in bed with 30 min compression point.
